# Stereotactic focal radiotherapy as an alternative treatment for low-risk prostate cancer: Results of a single-arm monocenter Phase-II trial

**DOI:** 10.3389/fonc.2023.1143716

**Published:** 2023-04-06

**Authors:** Paul V. Nguyen, Bertrand Donneaux, Céline Louis, Zsuzsa Bodgal, Sven Philippi, Sylvie Biver, Bérangère Frederick, Ludovic Harzé, Yves Lasar, Guillaume Vogin, Philippe Nickers

**Affiliations:** ^1^ Department of Radiotherapy, CHU UCL Namur – Site Saint Elisabeth, Namur, Belgium; ^2^ Department of Radiotherapy, Centre François Baclesse, Esch-sur-Alzette, Luxembourg; ^3^ Department of Radiology, Emile Mayrisch Hospital, Esch-sur-Alzette, Luxembourg; ^4^ Department of Radiotherapy, CHU de Liège, Avenue de l’Hopital 1, Liège, Belgium

**Keywords:** prostate cancer, stereotactic radiation, SBRT, focal therapies for prostate cancer, morbidity, Phase II trial, focal radiotherapy, focal SBRT

## Abstract

**Introduction:**

Since radical treatments in low risk prostate cancer do not improve overall survival in comparison to active surveillance, preserving quality of life (QOL) remains the key objective. Active surveillance of indolent prostate cancer avoids curative treatment side-effects but necessitates repeated biopsies. Focal stereotactic body radiation therapy (focal SBRT) may be an alternative. This non-randomized Phase-II trial examined the feasibility and safety of focal SBRT for low and favorable intermediate-risk prostate cancer.

**Methods:**

Patients were recruited in 2016–2019 if they had: localized CAPRA ≤ 3 prostate adenocarcinoma; an isolated PIRADS≥4 macroscopic tumor on MRI; WHO Performance Status 0-1; and no major urinary symptoms. 36.25 Gy (80% isodose prescription) were delivered in 5 fractions every other day. Primary outcome was delay between focal SBRT and salvage-treatment initiation. Secondary outcomes were: acute/late genitourinary/rectal toxicity; biological, clinical and MRI local control; and change in QOL measures.

**Results:**

Over a median follow-up of 36 months, salvage prostatectomy in the 24 eligible patients was never required. Three-year biochemical progression-free survival was 96%. The single biochemical recurrence was a small (2-mm) Gleason 6 (3 + 3) lesion in the non-irradiated lobe. All 19 patients with ≥1 post-treatment MRI evaluations demonstrated complete radiological response. Acute/late grade ≥3 toxicities did not occur: all acute toxicities were grade-1 genitourinary (38% patients), grade-2 genitourinary (8%), or grade-1 rectal (13%) toxicities. There was one (4%) late grade-1 genitourinary toxicity. QOL was unchanged at last follow-up, as shown by IPSS (2.86 to 3.29, p>0.05), U-QOL (0.71 to 0.67, p>0.05), and IIEF5 (the 14 initially potent patients maintained potency (IIEF5 > 16)).

**Conclusion:**

Focal SBRT is feasible, well-tolerated, and preserves QOL. This innovative robotized approach challenges active surveillance.

## 1 Introduction

Active surveillance of prostate cancer helps to avoid the morbidity associated with curative radical prostatectomy (RP)/radiotherapy in patients with indolent disease. Large patient cohorts with 20-year clinical follow-up also confirm that this approach is safe (1, 2). However, such monitoring requires repeated prostate ultrasounds and prostate biopsies. The latter are painful, can induce sepsis, and are still considered high-risk medical procedures (1, 2). Consequently, surveillance compliance is low: for example, a study on 4547 men showed that when patients were recommended to undergo repeat biopsies at 1, 4, 7, and 10 years, only 30% complied (1, 2). An alternative to repeat biopsies is to monitor prostate-serum antigen (PSA) alone; however, this approach is limited by the weak diagnostic accuracy of PSA. Yet another approach is to follow patients up with magnetic resonance imaging (MRI) and targeted biopsies, but this is still being researched (1, 2).

It should also be noted that patients who opt for active surveillance rather than initial definitive intervention base their decision on the notion that disease progression will be detected at a curable stage. However, a prospective cohort study of 993 men with favorable-risk prostate cancer undergoing active surveillance showed that of the 6% who demonstrated progression after some surveillance, a quarter could not be controlled (3). Moreover, the PROTECT Phase-III trial (which is often used to validate active surveillance) showed that at 10 years of follow-up, over 50% of the actively surveilled patients had required curative treatment. This was despite the fact that three-quarters of the actively surveilled group had started with an excellent prognosis (4). In addition, a Swedish study concluded that compared to watchful waiting, RP was associated with significantly lower rates of metastatic evolution that were independent of the better overall survival of younger patients (5).

These observations suggest that prostate-focused therapies that are intermediate in their aggressiveness may be useful: these approaches simultaneously destroy the index lesions, thereby reducing dissemination rates, while imposing much lower morbidity rates than RP/radiotherapy (6, 7). These advantages were recently illustrated by a Phase-III study on photodynamic therapy in low-risk prostate cancer: the treatment associated with both high tolerability and significantly less disease progression than active surveillance (8). The intermediate aggressive approach is also supported by the possibility that salvage RP is safer than previously, probably because the smaller tumor volumes permit the use of more conservative radiotherapy approaches: this view is currently widely held, although it remains to be proven by prospective trials (9).

A recent systematic review retained 72 studies on various focused therapies, including cryotherapy, high-intensity focused ultrasound, photodynamic therapy, and brachytherapy. Nearly all are at an early research stage and their long-term oncological effectiveness remains to be definitively determined but there is high-quality evidence showing that focused therapy associates with few adverse effects (6). However, it should be noted that all of these approaches require general anesthesia (sometimes repeated), which can itself associate with acute morbidity and reduced patient quality-of-life (QOL).

An alternative focused therapy may be prostate-focused stereotactic body radiation therapy (SBRT): this is a highly precise approach that does not require anesthesia and thus has the potential to prevent dissemination with very little morbidity. To determine the morbidity/benefit ratio of this approach, we conducted a prospective Phase-II clinical trial.

## 2 Materials and methods

### 2.1 Study design and ethics

This monocenter non-randomized Phase-II trial was conducted in the National Radiotherapy Center in Luxembourg. It was approved by the National Review Board of Luxembourg, and complied with the Declaration of Helsinki. All participants signed an informed consent form.

### 2.2 Patient recruitment, risk assessment, and follow-up

All consecutive patients who met the following inclusion criteria in February 2016–November 2019 were recruited prospectively: (i) a single low-risk localized prostate adenocarcinoma [defined as Cancer of the Prostate Risk Assessment (CAPRA) score (10) ≤3], (ii) an isolated macroscopic tumor [defined as Prostate Imaging Reporting and Data System (PIRADS) score ≥4 on multiparametric MRI], (iii) WHO Performance Status 0–1, (iv) no major urinary symptoms [defined as International Prostate Symptom Score (IPSS) ≤15]. Exclusion criteria were androgen-deprivation therapy, cancer within the last 5 years, prostatic transurethral resection, urethral stenosis, recurrent prostatitis, sigmoid diverticulitis, and any inflammatory collagen disease.

At diagnosis, all patients underwent MRI and systematic biopsies taking at least 12 biopsies with 6 samples in each lobe and additional biopsies in the MRI positive zone. MRI-targeted biopsies alone were not a prerequisite for inclusion but the positive biopsies had to match systematically the MRI lesions. Patients were followed up with PSA analyses at 3, 6, and 12 months and every 12 months thereafter. Prostate MRI was planned every 12 months to assess the radiological response until complete response was obtained.

### 2.3 SBRT

The SRBT procedure involved at first placement of two fiducial-marker strands (each containing two gold seeds), a planning computed tomography (CT) scan as well as a planning MRI, both fused thereafter based on fiducial markers. Rectal and bladder preparation were described in our study on whole prostate SBRT (11). SBRT was delivered as 36.25 Gy in 5 fractions prescribed on the 80% isodose or higher. The fractions were delivered with a CyberKnife M6™ every other day for 2 weeks.

**Figure 1:**
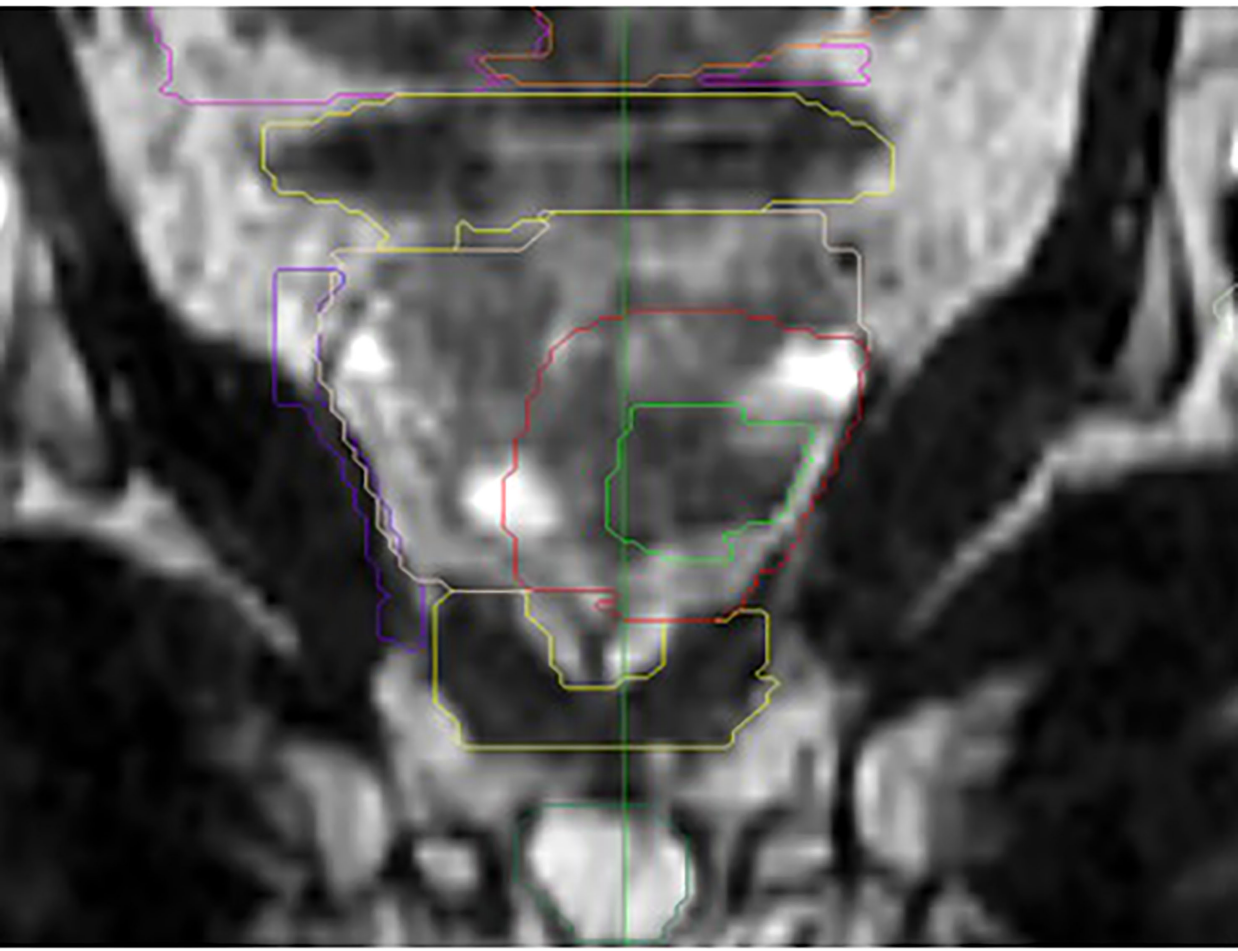
Example of delineated focused stereotactic treatment on the MRI T2 sequence frontal view. Purple = contralateral neurovascular bundle. Lower yellow structure = external sphincter. Light green = gross target volume. Red = clinical target volume. Dark green = penile bulb.

**Figure 2:**
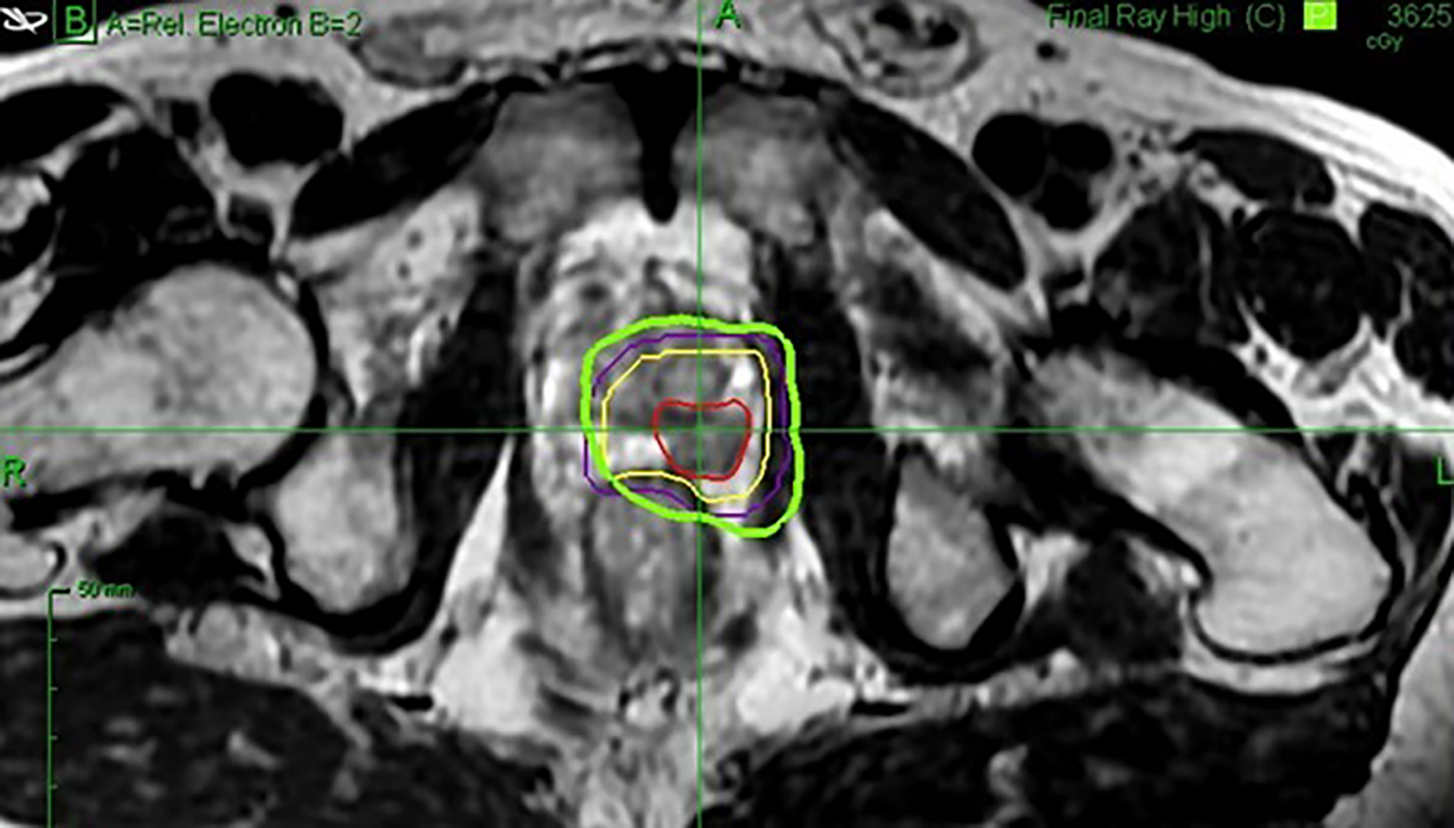
Example of a delineated focused stereotactic treatment on the MRI T2 sequence axial view. Red = gross target volume. Yellow = clinical target volume. Purple = planning target volume. Green = 36.25 Gy isodose.

Organs-at-risk (rectal wall, bladder wall, bladder neck, and urethra) and dose constraints were defined as we described previously (11). Contralateral neurovascular bundles and the contralateral external sphincter were specifically delineated for this study (**Figure 1**). A mean dose of <21 Gy was accepted for both. Gross target volume (GTV) was delineated on the fusion MRI while considering the hypointense T2-weighted nodule, the hypointense apparent diffusion coefficient, and the hyperintense perfusion zone visualized on the diagnostic multiparametric MRI. In all cases, the final GTV contour was validated by the same prostate cancer-imaging radiology specialist (YL). At first, the GTV was expanded by 1cm in all directions to generate the clinical target volume (CTV). The CTV was then cropped to the limits of the prostate. If the ratio CTV/Prostate was under 30%, the expansion margin of the GTV was increased progressively within the prostate by 0.1 cm increments so that the CTV achieved at least 30% of the prostate volume. Hence, a maximum of 1.5 cm margin around the GTV was allowed. The CTV was then expanded by 3 mm to generate the planning target volume (PTV) (**Figure 2**).

### 2.4 Primary and secondary study outcomes

The primary study outcome was time between treatment and salvage-treatment initiation. Secondary outcomes were: acute and late genitourinary and gastrointestinal (rectal) toxicity; biological, clinical, and radiological control; overall survival; and QOL. Recurrence was defined biologically by the Phoenix definition (12) or radiologically by the appearance of a suspicious nodule on MRI that necessitated biopsy. Toxicity was graded according to the Common Terminology Criteria for Adverse Events version 4. QOL was measured with the IPSS score, the patient-reported Urinary QOL (U-QOL) questionnaire, and the International Index of Erectile Function (IIEF)5 Scale. Potency was defined as IIEF5 > 16.

### 2.5 Statistical analyses

Normally and non-normally distributed continuous variables were expressed as mean ± standard deviation (SD) and/or median (range), respectively. Categorical variables were expressed as *n* (%). Change in IPSS, U-QOL, and IIEF5 at last visit relative to baseline was assessed with Mann-Whitney U test. All statistical analyses were conducted with Microsoft Excel Version 1808. P values <0.05 indicated statistical significance.

## 3 Results

### 3.1 Baseline and treatment characteristics of the cohort

In total, 24 patients were recruited prospectively from February 2016 to November 2019. Median follow-up duration was 36 (6–48) months. Only one patient was lost to follow-up: he declined to continue participating 6 months after study entry due to moving to another continent. The baseline characteristics of these patients are shown in **Table 1**. Median age at diagnosis was 66 (range: 55–79) years. Median pretreatment PSA was 7.08 (2.4–13.0) ng/ml. CAPRA-based risk indicated that 15 (62.5%) and 9 (37.5%) patients had low and favorable intermediate risk prostate cancer, respectively. Each patient harbored an MRI PIRADS 4-5 score nodule and thus a macroscopic disease.

**Table 1:** Baseline characteristics of the patients.

Number of patients	24
Median age, years	66 (55 – 79)
Gleason score 6 7 (3 + 4)	22 (91.7%) 2 (8.3%)
Clinical stage T1c T2a T2b	12 (50%) 9 (37.5%) 3 (12.5%)
PSA level, ng/ml Median PSA <10 ≥10	7.08 (2.4–13.0) 19 (79.2%) 5 (20.8%)
Percent biopsy cores positive for cancer < 34% ≥ 34%	22 (91.7%) 2 (8.3%)
Risk group, CAPRA score 1 2 3	7 (29.2%) 8 (33.3%) 9 (37.5%)
Median treatment time, days	10 (9 – 13)

The data are expressed as median (range) or n (%), as appropriate.

CAPRA, Cancer of Prostate Risk Assessment; PSA, prostate serum antigen.

Median overall treatment time was 10 (9–13) days. The dose volume histogram variables are presented in **Table 2**. Mean prescribed isodose was 82.4 ± 3.3%. Prostate GTV and CTV volumes were 6.01 ± 8.9 cc, and 20.5 ± 10 cc, respectively. The CTV volume accounted for 34.0 ± 8.7% of prostate volume on average. The D98, D50, and D2 of the PTV were 35.5 ± 0.9, 40.0 ± 0.9, and 43.0 ± 1.5 Gy, respectively. All patients met the rectum and bladder dose-constraint criteria. Mean contralateral neurovascular bundle and urethral sphincter doses were 15.4 ± 6.9 and 15.3 ± 3.7 Gy, respectively.

**Table 2:** Dose volume histogram variables of the patients.

	Focal SBRT (36.25, 5x7.25 Gy)	
Prescribed isodose	82.4 ± 3.3%
Volume, cc		
Prostate GTV CTV Ratio CTV/Prostate	61.0 ± 23.5 6.01 ± 8.9 20.5 ± 10 34.0 ± 8.7%
DVH, Gy		
GTV D2% D50% D98%	43.0 ± 1.8 41.0 ± 1.5 38.6 ± 1.5
CTV D2% D50% D98%	43.2 ± 1.5 41.0 ± 1.1 37.8 ± 0.7
PTV D2% D50% D98%	43.0 ± 1.5 40.0 ± 0.9 35.5 ± 0.9
Rectal wall V36.25 V27 V23 V20 V35	1.2 ± 0.7% 10.9 ± 4.7% 14.1 ± 5.4% 16.8 ± 6.2% 0.8 ± 0.5cc
Bladder Wall V36.25 V27 V23 V20 V35	0.3 ± 0.4% 5.5 ± 4.9% 9.4 ± 6.6% 14.2 ± 7.9% 0.4 ± 0.6 cc
Bladder neck V35	0.1 ± 0.2 cc
Anal canal V36.25 <8% V27 <20%	0.1 ± 0.4% 2.6 ± 3.4%
Membranous urethra D0.04 cc	32.1 ± 7.4
Prostatic urethra D0.04 cc	37.6 ± 3.3
Bulb D0.04 cc	6.6 ± 4.2
Neurovascular bundle Dmean D0.04 cc	15.3 ± 3.7 26.4 ± 8
Urethral sphincter Dmean D0.04 cc	15.4 ± 6.9 27.8 ± 10.1

GTV, gross target volume; CTV, clinical target volume; DVH, dose-volume histogram; PTV, planning target volume; Dmean, Mean dose; Neurovascular bundle, Controlateral Neurovascular bundle; Urethral sphincter, Controlateral urethral sphincter. Mean.

### 3.2 Biological and radiological control during follow-up

Median PSA nadir was 0.96 (0.32–4.73) ng/ml and median time to nadir was 27 months. By 36 months, neither salvage RP nor other rescue treatments had been performed. The 3-year biochemical progression-free survival rate of the cohort was 96% (23/24).

The sole patient who experienced a biochemical recurrence did so at 24 months. This was accompanied by MRI showing a PIRADS 4 nodule in the contralateral lobe that fell outside the irradiated area. The previously treated nodule did not display recurrence. Biopsies did not find any cancer signs in the treated area but did detect a small 2 mm-long Gleason 6 (3 + 3) adenocarcinoma in the contralateral lobe. At the time of the data analysis, the PSA stabilized later on, so no treatment was required yet.

The 3-year overall survival of the whole cohort was 96% (23/24). The patient who died had a myocardial infarction at 37 months.

Five patients declined post-treatment MRI. Of the 19 (79%) patients who underwent a follow-up MRI, complete radiological responses were found in all but one. The exception was the patient with biochemical failure and a MRI-detected lesion in the contralateral lobe described above. Hence, the MRI local control of the treated lesions was 100%.

### 3.3 Acute, late toxicity and quality-of-life during follow-up

The treatment was overall well tolerated. None of the patients experienced acute or late severe grade 3-4 toxicity. However, acute grade 1-2 genitourinary (GU) and gastrointestinal (GI) toxicity were observed in 11 (46%) and 3 (13%) patients, respectively. Of these, 2 (8%) experienced grade 2 genitourinary toxicity (dysuria and urgency). GI tolerance was good since acute or late grade 2 proctitis was never observed. Only one patient (4%) reported a late toxicity, namely, a grade 1 GU toxicity. Cumulative incidence of GU and GI toxicity and adverse events are presented in **Table 3**.

**Table 3:** Cumulative acute and late toxicity.

Acute toxicity	Grade 0	Grade I	Grade II	Grade ≥ III
**Genitourinary** *Dysuria* *Urgency* *Incontinence* *Hematuria* **Gastrointestinal** *Proctitis*	13 (54.2%) 14 (58.3%) 23 (95.8%) 24 (100%) 24 (100%) 21 (87.5%)	9 (37.5%) 9 (37.5%) 0 0 0 3 (12.5%)	2 (8.3%) 1 (4.2%) 1 (4.2%) 0 0 0	0 0 0 0 0 0
Late toxicity	Grade 0	Grade I	Grade II	Grade ≥ III
**Genitourinary** *Dysuria* *Urgency* *Incontinence* *Hematuria* **Gastrointestinal** *Proctitis*	23 (95.8%) 23 (95.8%) 24 (100%) 24 (100%) 24 (100%) 24 (100%) 24 (100%)	1 (4.2%) 1 (4.2%) 0 0 0 0 0	0 0 0 0 0 0 0	0 0 0 0 0 0 0

**Figure 3:**
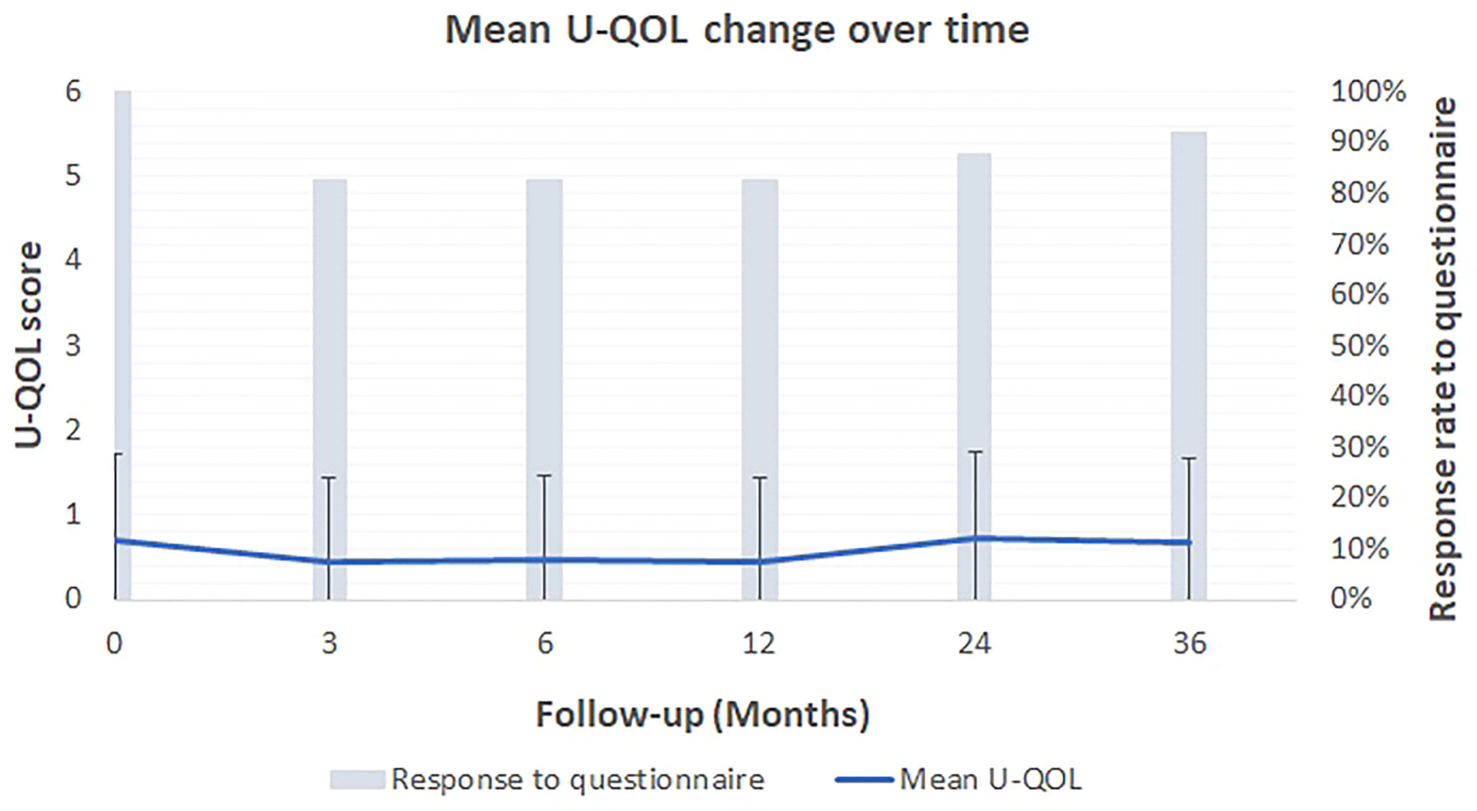
Patient-reported outcome: Mean U-QOL score change over time along with the standard deviation from the mean score.

**Figure 4:**
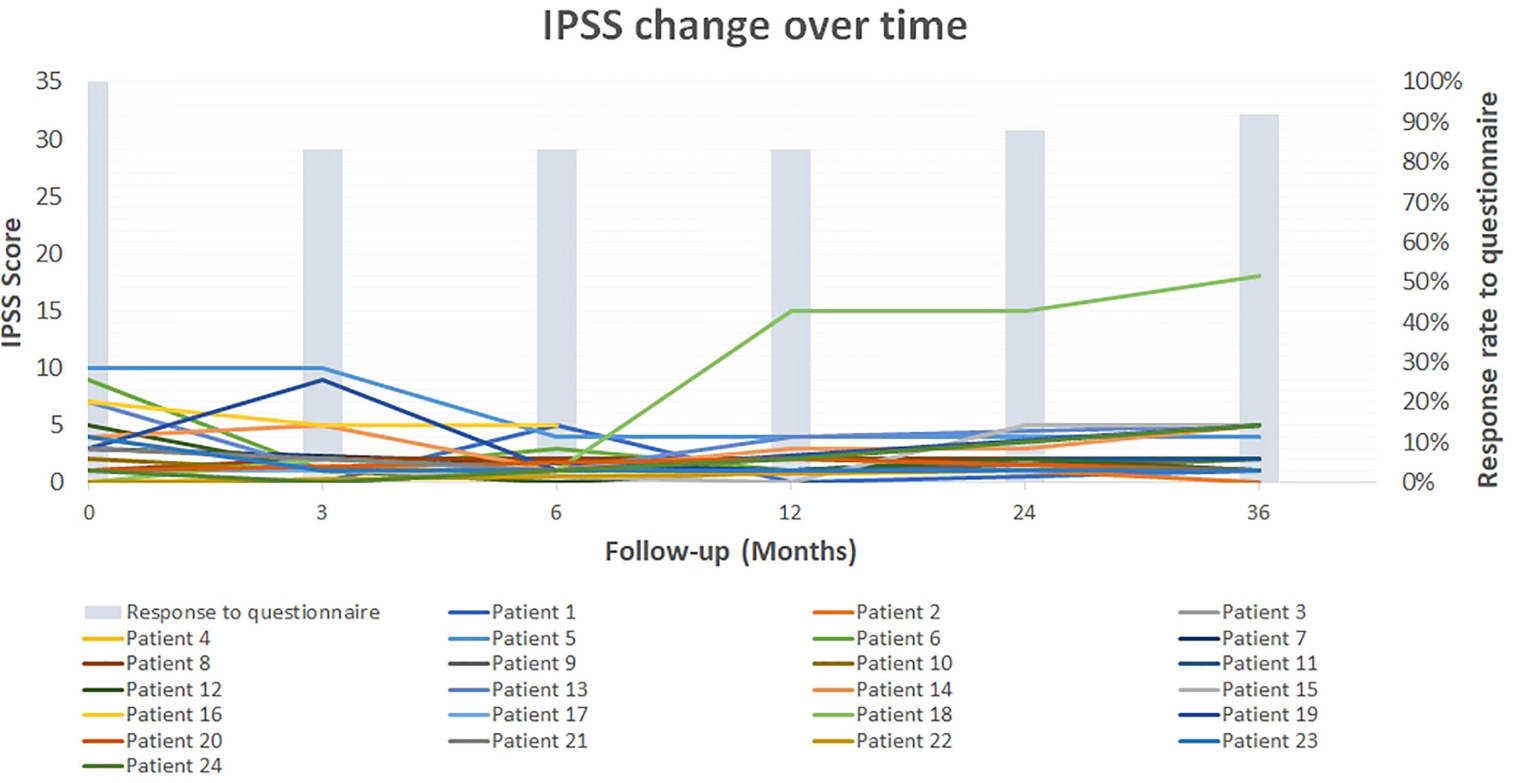
Patient-reported outcome: Change in IPSS score over time for the 24 patients. Patient 18 suffered an accidental trauma to the lumbar spine causing a neurogenic bladder.

The IPSS, U-QOL and IIEF5 scores did not worsen significantly after treatment (p>0.05) (**Table 4**). The mean U-QOL change over time is presented in **Figure 3**. At baseline and the end of follow-up, the mean IPSS were 2.90 ± 2.84 and 3.29 ± 3.83, respectively. **Figure 4** shows the IPSS change over time for the 24 patients. It should be noted that one patient reported an IPSS score from 0 to 18. Indeed, this patient suffered an accidental trauma to the lumbar spine causing a neurogenic bladder. These urinary symptoms have not been registered as related to our treatment. Apart from this patient, the highest IPSS score at the end of the follow-up was 5 and was reported by 5 patients.

**Table 4:** Quality of life indicators.

Variable			
	Baseline	Last FU	P value*
IPSS	2.90 ( ± 2.84)	3.29 ( ± 3.83)	p>0.05
U-QOL	0.70 ( ± 0.69)	0.67 ( ± 0.92)	p>0.05
IIEF-5	16.1 (± 9.08)	17.0 (± 9.27)	p>0.05

*P values for change relative to baseline, as determined by Mann-Whitney U test.

FU, follow-up; IIEF-5, International Index of Erectile Function-5 Scale; IPSS, International Prostate Symptom Score; U-QOL, Urinary Quality of Life questionnaire.

Of the 14 patients (58.33%) who were initially potent, erectile function could be well preserved since all remained potent at last follow-up.

## 4 Discussion

This study showed that over 36 months, 24 patients with a low and favorable intermediate risk (CAPRA-score 1–3) but macroscopic (PIRADS 4-5) prostate adenocarcinoma who underwent prostate-focal SBRT exhibited good tolerance to SBRT: acute toxicities were mild, late toxicity was rare and mild, IPSS and U-QOL scores did not change, and erectile capacity was unaffected. Moreover, focal SBRT associated with complete MRI response: all initially visualized nodules in the 19 patients who underwent repeat MRI disappeared. This was paralleled by marked PSA reductions (7.08 to 0.96 ng/ml at nadir). Furthermore, rescue treatment was never needed during 36-month follow-up: consequently, the primary study objective (median time to rescue RP) could not be measured. Only one patient demonstrated biochemical failure. The new tumor was small (2 mm) and located in the unirradiated contralateral lobe at 24 months.

The SBRT consisted of five 25-minute sessions delivered over 2 weeks. The only invasive part of the treatment that had potential to induce morbidity was placement of two fiducial-marker strands in the prostate *via* trans-perineal punctures under local anesthesia. Future SBRT devices will probably not require this step. Thus, SBRT is a quick procedure that does not require general anesthesia, associates with minimal short- and long-term morbidity, and may exert good oncological control. These points seem important given that interventions in the prostate cancer population do not appear to improve overall survival (4). Our good outcomes may reflect the fact that SBRT is not only non-interventional but also robotized: it does not require manual human intervention during the procedure and integrates MRI data due to fusion with the CT scans used for planimetry. These elements naturally increase the precision of the procedure.

Thus, we propose that focal prostatic SBRT may be a good intermediately aggressive treatment that could significantly reduce metastatic dissemination while imposing only mild morbidity. This approach could therefore help avoid the uncontrollable recurrences that arise during watchful waiting or active surveillance (3). Moreover, given that all other published focal treatments (6) must be performed under general anesthesia, the low interventional nature of SBRT suggests that it may be a suitable alternative to these treatments as well. In the future a cost-benefit approach would certainly deserve to be realized. However, such a work has to be conducted in a prospective study comparing active surveillance and focused treatments.

The stereotactic whole prostate radiotherapy has been monitored for long-term, considering prostatic tumors with good prognosis or intermediate prognosis from 12 prospective studies (13). Out of the 2142 patients treated, 1185 had a disease with a good prognosis, 692 with a favorable intermediate prognosis and 265 with an unfavorable intermediate prognosis. At a median follow-up of 6.9 years, biochemical recurrence rates for these prognostic families were 4.5%, 8.6% and 14.9% respectively. The rates of late genitourinary complications of grade 3 or higher were 2.4% while the rates of gastro-intestinal complications of grade 3 or higher were 0.4%. Compared to other prostatic radiotherapy techniques, stereotactic radiotherapy was located in radiotherapy techniques causing the least late morbidity. Moreover, the two Phase III trials on whole prostate SBRT, namely HYPO-RT-PC and PACE-B, corroborate these good oncological results and toxicity profile (14, 15). These data therefore suggest that stereotactic radiotherapy is an appropriate curative treatment for prostatic tumors with good or intermediate prognosis (13–15).

In addition, modern external non-stereotactic radiation therapy or robotic surgery techniques have recently been compared to active monitoring for quality of life data (16). Out of 1386 cancer patients with a favorable prognosis, external radiation therapy without hormone therapy was not more deleterious than active surveillance, including for the progressive loss of erectile function over time. It should be noted that the toxicity inherent in repeated biopsies in case of active surveillance cannot be taken into account in these comparisons. The same applies to the 25% of patients on active surveillance arms who will be treated curatively within five years with inherent added toxicity (16).

Several articles have recently been published on the various techniques of focused prostate therapies (6, 7). Few radiotherapy techniques are mentioned except brachytherapy. However, the latter technique requires general anesthesia like the other focused treatment techniques. We can question indeed on the heaviness of general anesthesia, sometimes repeated, for focused therapy that wants to compete with active surveillance. In contrast, stereotactic radiotherapy does not impose any anesthesia while inducing particularly low toxicity (13). Partial prostate irradiation should also reduce toxicity given the smaller volumes of irradiated tissues. Finally, focal SBRT in comparison to all the other published methods seems by far the less invasive.

It should be noted that prostatic cancers at the macroscopic stage have a higher metastatic potential than smaller cancers (17). While the merits of focused therapy for localized prostate cancer are still being debated (18), it is possible that they could be even more effective for more aggressive tumors (*e.g.* Gleason score ≥7 (3 + 4) tumors). Genetic tests and/or other innovative prognostic factors will aid the selection of the populations that will benefit the most from focused treatment. Notably, the ASCO GU ​​​​2022-2023 has examined the conditions of active monitoring in such situations, which supports the notion that focused therapies might serve an alternative. Thus, new Phase-II and -III trials on the role of focused SBRT in prostate cancer may illuminate new treatment possibilities.

Study limitations include the small sample size, which reflects the exploratory nature of this study. Its relatively short follow-up (median 36 months) is also a study limitation.

## Data availability statement

The raw data supporting the conclusions of this article will be made available by the authors, without undue reservation.

## Ethics statement

The studies involving human participants were reviewed and approved by Centre National du Cancer Luxembourgeois. The patients/participants provided their written informed consent to participate in this study. Written informed consent was obtained from the individual(s) for the publication of any potentially identifiable images or data included in this article.

## Author contributions

PN conceived the project and designed the study. All contributors performed the data extraction and collection. PN and PVN performed the data analysis. PN and PVN wrote and revised the manuscript. All authors contributed to the article and approved the submitted version.
